# Cellular Senescence in Germ Cell Neoplasia In Situ (GCNIS) and Other Histological Types of Testicular Cancer

**DOI:** 10.3390/medicina60071108

**Published:** 2024-07-08

**Authors:** Vasileios Tatanis, Dimitris Veroutis, Pavlos Pantelis, George Theocharous, Helen Sarlanis, Alexandros Georgiou, Francesk Mulita, Angelis Peteinaris, Anastasios Natsos, Napoleon Moulavasilis, Nikolaos Kavantzas, Athanassios Kotsinas, Ioannis Adamakis

**Affiliations:** 1 Department of Urology, University of Patras, 26504 Patras, Greece; tatanisbas@gmail.com (V.T.); peteinarisaggelis@gmail.com (A.P.); a.natsos@gmail.com (A.N.); 2 Molecular Carcinogenesis Group, Department of Histology and Embryology, Medical School, National Kapodistrian University of Athens (NKUA), 11527 Athens, Greece; dimitrisveroutis1@gmail.com (D.V.); pavlospantelis7@gmail.com (P.P.); theocharousgiorgos@gmail.com (G.T.); akotsin@gmail.com (A.K.); 3 Department of Pathology, Medical School, National and Kapodistrian University, 10680 Athens, Greece; elenisarlani@gmail.com (H.S.); alexandrosge11@gmail.com (A.G.); nkavantz@med.uoa.gr (N.K.); 4 Department of Surgery, University Hospital of Patras, 26504 Patras, Greece; 5 1st Department of Pathology, School of Medicine, National and Kapodistrian University of Athens, 10680 Athens, Greece; napomoul@hotmail.com (N.M.); yianton@hotmail.com (I.A.)

**Keywords:** germ cell neoplasia in situ, GL13, immunohistochemistry, senescence, testicular cancer

## Abstract

*Background and Objectives:* The presence and contribution of senescent cells in premalignant lesions is well documented, but not in germ cell neoplasia in situ. The purpose of this study is to identify the presence of senescent cells in pre-malignant testicular conditions and in different histological types of testicular cancer. *Materials and Methods:* Thirty patients who underwent orchiectomy due to testicular tumors were included. Formalin-fixed paraffin-embedded (FFPE) testicular tissue for each patient was available. Sections from these specimens were examined by immunohistochemical analysis with the following markers: GL13 for cellular senescence, p21^WAF1/Cip1^ for cell cycle arrest, and Ki67 for cell proliferation. *Results:* Thirteen (43.3%) suffered from seminoma with a mean total proportion of GCNIS senescence of 20.81 ± 6.81%. In the group of embryonal testicular tumors, nine (30%) patients were included, with an average rate of 6.64 ± 5.42% of senescent cells in GCNIS. One (3.3%) patient suffered from chondrosarcoma in which 7.9% of GL13+ cells were detected in GCNIS. Four (13.4%) patients suffered from teratoma and three (10%) from yolk sac tumors, while GCNIS senescence was detected in a range of 4.43 ± 1.78% and 3.76 ± 1.37%, respectively. *Conclusions:* Cellular senescence was detected in both germ cell neoplasia in situ and testicular cancer, but was more prevalent within the premalignant lesions.

## 1. Introduction

Testicular tumors constitute an urological oncologic entity that refers mainly to adults younger than 50 years old [1]. Germ cell tumors (GCTs) represent the most common type of testicular tumor (90–95%), including a variety of different histological subtypes, which are stratified into two main groups: tumors that originate from germ cell in situ neoplasia (GCNIS) and non-GCNIS tumors.

GCNIS constitutes a major precancerous situation. It encompasses the old terms of intratubular germ neoplasia, and it accompanies the majority of GCTs (over 70%) and 24% of embryonal carcinomas [2,3]. GCNIS is considered to be the result of the failure of the differentiation of the gonocytes (immature germ cells) to the spermatogonia [4]. Regarding morphology, GCNIS cells are similar to fetal gonocytes, although the chromatin in the nuclei seems to be more unusual. The most common location of GCNIS cells is inside seminiferous tubules, which may be well developed or hypoplastic, forming a single row adjacent to the basement membrane in most cases [5,6].

Cellular senescence is a stress response condition in the context of homeostasis [7]. Senescent cells are characterized by irreversible growth arrest, during which they remain metabolically active and exhibit secretory characteristics [8]. The latter is known as the senescence-associated secretory phenotype (SASP), which is a hallmark of senescence, and depending on the type of senescence and cells participating, a range of cytokines, chemokines, growth factors, proteases, and other factors are released by these cells. Cellular senescence may act both as a barrier and as an inducer of carcinogenesis [9]. In early premalignant stages, it serves as an antitumor barrier to prevent further malignant transformation [10]. Nevertheless, if senescent cells are not eliminated in a timely manner, they may either promote tumor development via SASP or escape from this condition [10]. In both cases, emerging tumors exhibit more aggressive features, providing a potential explanation for tumor recurrence. Collectively, the above suggests that monitoring senescence can be a valuable tool during cancer development and a potential target to improve therapeutic efficacy [7,11].

The presence of cellular senescence in premalignant conditions of visceral tumors has been widely documented. Similarly, their occurrence has been shown in low-grade pancreatic intraepithelial lesions that act as precancerous lesions of pancreatic tissue [12]. To our knowledge, cellular senescence has not been evaluated in testicular cancer. The purpose of this study is to identify its presence and the population of such cells in pre-malignant and tumor testicular conditions of different histological types.

## 2. Materials and Methods

### 2.1. Statement of Human Rights

The study was carried out in accordance with the ethical standards laid down in the 1964 Declaration of Helsinki and its later amendments. Informed consent was obtained from all included patients.

### 2.2. Study Design

This retrospective experimental study comprised patients who underwent orchiectomy due to testicular tumors between 2011 and 2019 at the 1st Urology Department of Athens Medical School. The age under 18 years, orchiectomy for secondary tumors, application of preoperative chemotherapy, incomplete preoperative data, and loss during follow-up were defined as exclusion criteria. Formalin-fixed paraffin-embedded (FFPE) testicular tissues harboring cancerous and normal material were collected from the histopathological laboratory. Sections of 4 μm comprising concomitantly normal, premalignant (if present), and malignant tissue, were obtained from each sample (patient) followed by immunohistochemical (IHC) analysis. Specifically, GL13, p21^WAF1/CIP1^ and Ki-67 staining were applied. GL13 (SenTraGor trademark) staining constitutes an extremely efficient and broadly known marker for senescent cell identification, whereas p21^WAF1/CIP1^ is a protein that is rarely present in cases of normal testicular tissue or GCT [13,14]. Ki-67 is a proliferation marker that is strongly associated with the growth fraction of cells [15]. All three markers were assessed based on an algorithm for senescence detection proposed in the guideline manuscript by the International Cell Senescence Association (ICSA—https://www.cellsenescence.info/, accessed on 25 April 2024) [7,16]. A total of 30 cases were finally selected for analysis based on the quality of the FFPE material and data collection availability.

### 2.3. Data Collection

The demographic variables that were recorded included age of the patient and date of the operation. The size of the testis was recorded based on the histopathological report, as it was considered to be the most accurate measurement [17]. Regarding the tumors, size, histopathological type, and pathological stage were recorded. In the case of histopathologically mixed tumors, all the included types and the corresponding rates were recorded. Patients were stratified into groups based on the primary histological diagnosis. Additionally, the presence of lymphovascular invasion or embolism (LVI), the invasion of rete testis (RTI), and the presence of GCNIS were noticed.

### 2.4. Immunohistochemistry for Anti-Biotin, p21^WAF1/CIP1^, and Ki67 Staining

Sections from the FFPE samples were deparaffinized and hydrated and antigen retrieval was heat mediated using 10 mM of citric acid (pH 6.0) for 15 min in a steamer (in the case of SenTraGor^TM^ staining). For sections that were incubated with p21^WAF1/CIP1^ and Ki67 antibodies, antigen retrieval was completed in the same way, but with a heating step of 25 min in the microwave. Blocking of non-specific binding for the aforementioned antibodies was performed by applying normal goat serum for 1 h at room temperature (dilution 1:40, Abcam, Cambridge, UK ab138478). The following primary antibodies were applied: anti-biotin antibody (dilution 1:300, Hyb-8, ab201341, Abcam), p21^WAF1/Cip1^ (dilution 1:200, 12D1, Cell Signaling, Danvers, MA, USA), and Ki-67 (dilution 1:250, Sp-7, Abcam), and tissues were incubated overnight at 4 °C. Signal development was performed with the Dako REAL EnVision Detection System kit (Cat.no: K5007) according to the manufacturer’s instructions using DAB as a substrate (brown color—end signal). Sections were counterstained with hematoxylin or Nuclear Fast Red and positive cells were countered. Finally, observations were performed using a ZEISS Axiolab5 optical microscope with a 20× objective (200× magnification, 25 μM scale bar) [16]. Counting was performed independently by three observers.

### 2.5. SenTraGor^TM^ (GL13) Staining for Senescence Detection

For SenTraGor^TM^ staining after the blocking step, sections were incubated in 50% ethanol for 5 min and then in 70% ethanol for an additional 5 min. SenTraGor^TM^ was applied on tissues and the sections were covered with glass coverslips. Samples were incubated at 37 °C for 10 min. Following that, coverslips were gently removed, and sections were washed with 50% ethanol for 30 s. An extra wash step was performed (3–5 min) using Triton-X 0.3%/TBS. Sections were washed with TBS and the anti-biotin antibody (dilution: 1/300, Cat.no: K5007) was applied overnight at 4 °C [16]. Counting was performed independently by three observers.

### 2.6. Statistical Analysis

Continuous variables were examined for mean values and standard deviation. Categorical variables were tested as absolute numbers and rates. The two-way *t*-test and two-way z-test were used for the comparison of continuous variables between groups, while the Chi-square test was utilized for categorical variables. Statistical analysis was conducted utilizing the Statistical Package for the Social Sciences (SPSS) software package version 25.0 (IBM Corp., Armonk, NY, USA).

## 3. Results

In this study, we interrogated samples obtained from 30 patients to assess the presence and frequency of senescent cells in normal tissue, pre-malignant conditions, and testicular tumors of different histological types. Notably, we obtained this sequel of histological stages, whenever available, from each patient. The mean age of the patients was calculated to be 30.97 ± 8.68 years, while the mean maximum diameter of the testis was estimated to be 6 ± 2.80 cm. Finally, the mean maximum diameter of the tumor was 4.16 ± 3.25 cm (Table 1).

**Figure 1:**
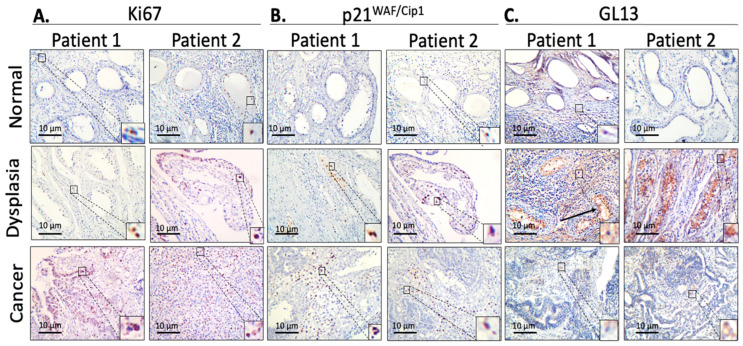
Evaluation of cell proliferation and cellular senescence via immunohistochemistry (IHC) staining in normal, dysplastic/GCNIS, and neoplastic (cancerous) testicular tissue, from a tissue biopsy of a single patient. Marker positivity was visualized through DAB staining and is indicated by a brown color. Representative images of two anonymized patients of (**A**) Ki67 (cell proliferation), (**B**) p21^WAF1/Cip1^, and (**C**) GL13 staining (cellular senescence). Scale bar 10 μm.

**Figure 2:**
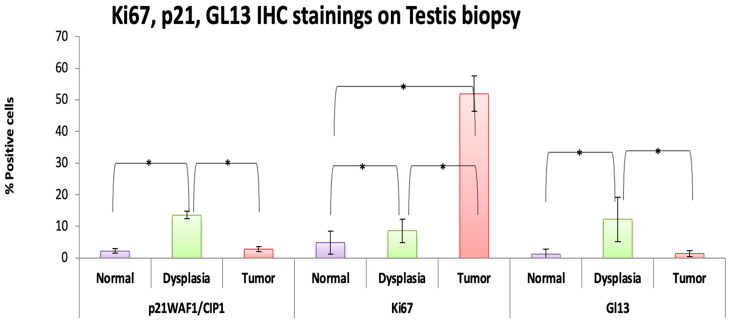
Comparative study showing the number of positive cells for the examined biomarkers in normal testicular tissue, dysplastic tissue/GCNIS, and neoplastic testicular tissue. Corresponding graphs depicting quantification of GL13, p21^WAF1/Cip1^ and Ki-67 in the normal, pre-cancerous, and cancerous tissues of testicles. (Statistically significant differences are marked with *).

The presence of Ki67+, p21^WAF1/CIP1^ (+), and GL13(+) cells was evaluated in normal testicular tissue, GCNIS, and testicular neoplastic tissue (independently of the histology). Our analysis showed that in the normal tissue the frequency of these markers was minimal (p21^WAF1/CIP1^: 2.26 ± 1.14%, Ki67: 4.95 ± 1.93%, and GL13: 1.18 ± 0.90%), whereas cells positive for p21^WAF1/CIP1^ (13.62 ± 9.74%, *p* < 0.001) and GL13 (12.24 ± 9.66%, *p* < 0.001) were significantly increased in dysplastic testicular structures. While Ki-67 was slightly increased (8.61 ± 3.70%) in the latter tissues, the difference was statistically significant (*p* < 0.001). Regarding the malignant tissues, Ki-67 expression was found in about 51.96 ± 15.28% of the cells, while the presence of the other markers was minimal (p21^WAF1/Cip1^: 2.84 ± 1.22% and GL13: 1.36 ± 1.28%), almost similar to the level of normal tissues (Figure 1 and Figure 2).

**Figure 3:**
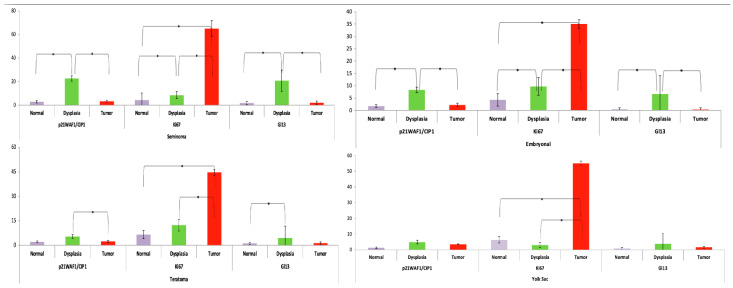
The proportion of p21^WAF1/CIP1^(+), Ki67+, and GL13(+) cells in different histopathological groups. (Statistically significant differences are marked with *).

Thirteen (43.3%) out of the 30 examined patients suffered from seminoma (Table 1). GCNIS was present in 11 of the 13 patients (84.6%). Notably, p21^WAF1/Cip1^ (+) cells were statistically increased in the dysplastic tissues (22.68 ± 5.83%), in contrast to the low levels in normal (2.97 ± 1.06%, *p* < 0.001) and tumor counterparts (3.32 ± 1.35%, *p* < 0.001). Ki67 positivity was mainly detected in cancer cells (78.08 ± 8.19%), while it was reduced in normal and dysplastic tissues (2.45 ± 0.89% and 9.56 ± 2.89%). The difference between tumor and normal or dysplastic tissues was statistically significant (*p* < 0.001 and *p* < 0.001, respectively) (Table 2). Ki67 staining was also significantly increased in dysplastic relative to normal tissues (*p* = 0.004). Concerning GL13(+) cells, they were predominately detected in GCNIS (20.81 ± 6.81%), with significantly lower frequency in malignant (2.07 ± 1.49%, *p* < 0.001) and normal counterparts (1.86 ± 0.84%, *p* < 0.001) (Table 2 and Table 3) (Figure 3).

In the group of embryonal testicular tumors, nine (30%) patients were included (Table 1). The presence of GCNIS was reported in seven (77.7%) patients. In this histological group, p21^WAF1/CIP1^ (+) cells were more commonly detected in the dysplastic tissues (8.29 ± 6.92%), and their presence reached statistical significance compared to normal (1.76 ± 1.08%, *p* = 0.02) and tumor counterparts (2.19 ± 1.11%, *p* = 0.04). Ki67(+) cells were predominately detected in tumors (35 ± 7.77%), while their presence was reduced in normal and dysplastic tissues (4.23 ± 1.09% and 9.67 ± 2.40%). All differences reached statistically significant levels (*p* < 0.001 each). Regarding the GL13(+) cells, the highest levels were detected in GCNIS (6.64 ± 5.42%), followed by decreased levels in normal (0.47 ± 0.43%) and cancerous counterparts (0.35 ± 0.18%). These differences between GCNIS versus normal and malignant counterparts were also statistically significant (*p* = 0.03 and *p* = 0.026, respectively (Table 2 and Table 4) (Figure 3).

One (3.3%) patient suffered from chondrosarcoma while GCNIS was present. Within the normal tissue, we observed 1.2% p21^WAF1/Cip1^ (+) cells, 7.5% Ki67 (+) cells, and 0.2% senescent cells. In GCNIS, the proportions were proven to be 3%, 2%, and 7.9%, respectively. Moreover, malignant tissues exhibited 54% Ki67+ cells, 2.5% p21^WAF1/CIP1^ (+) cells, and 1% GL13(+) cells (Table 1).

The histological type of teratoma was detected in four (13.4%) cases. In this group, we found the presence of 5.4 ± 2.51%, 2.13 ± 0.55%, and 1.33 ± 0.70% p21^WAF1/CIP1^(+) cells in GCNIS, normal, and tumor structures, respectively. The difference between GCNIS and malignant tissues was statistically significant (*p* = 0.011). The Ki67(+) cells were mainly abundant in tumor areas (44.70 ± 7.39%), and were significantly reduced in normal and dysplastic tissues (6.53 ± 1.16%, *p* = 0.002 and 12.30 ± 3.54%, *p* = 0.007). GL13(+) cells were predominately detected in GCNIS (4.43 ± 1.78%), followed by tumor (1.33 ± 0.7%) and normal counterparts (1.15 ± 0.68%). The difference between GCNIS and normal tissues reached a statistically significant level (*p* = 0.022) (Table 2 and Table 5) (Figure 3).

The histological diagnosis of the yolk sac tumor revealed three (10%) cases. In this group, the p21^WAF1/CIP1^ (+) cells were increased in the dysplastic tissues (4.9 ± 1.95%) compared to normal (1.27 ± 0.64%) and tumor counterparts (3.53 ± 0.72%), but this difference was not statistically significant. The Ki67(+) positivity was mainly detected in cancer cells (55 ± 6.56%) and was significantly reduced in normal and dysplastic tissues (6.33 ± 1.16%, *p* = 0.008 and 2.97 ± 1.51%, *p* = 0.007, respectively). The GL13(+) cells were predominately detected in GCNIS (3.76 ± 1.37%) followed by tumor (1.53 ± 0.55%, *p* = 0.19) and normal counterparts (0.77 ± 0.25%, *p* = 0.62) (Table 2 and Table 6) (Figure 3).

Taking into consideration recent reports proposing that testicular cancer sequentially progresses from normal tissues to GCNIS, followed by seminoma and finally by embryonal carcinoma [18], a comparison for the presence of senescent cells was conducted between these groups. The difference was statistically significant, particularly for the GCNIS and malignant counterparts (GCNIS: 20.82 ± 6.82% vs. 6.64 ± 5.42%, *p* < 0.001; tumor: 2.07 ± 1.49% vs. 0.35 ± 0.18%, *p* = 0.002), with the seminomas exhibiting higher levels of senescent cells compared to the embryonal carcinomas (Table 7). The other tumor types (chondrosarcoma, teratoma, and yolk sac tumor) were not further considered due to the low number of cases available.

## 4. Discussion

The aim of this study was to evaluate the presence of senescence, a cell reaction to stress, in normal and pre-malignant conditions and in various histological types of testicular cancer. Given that in a previous study we interrogated various clinical markers in testicular cancers, demonstrating that senescence was related to tumor size and the preoperative level of lactate dehydrogenase (LDH) [19], we sought to expand our analysis in tissue sections that harbored these progression stages for every analyzed patient. Sections were stained with GL13 to detect lipofuscin, an established biomarker of senescence [9]. Senescent cells stained with GL13 exhibited a pattern that was mainly perinuclear but also extended into a larger part of the cytoplasm (Figure 1c). This evaluation was complemented by assessing p21^WAF1/Cip1^ and Ki67 immunostaining, following an established multi-marker approach for the detection of senescent cells in situ [7].

The results of this investigation revealed that cellular senescence is strongly present in the GCNIS precancerous stage (concurrent assessment of GL13 and p21^WAF1/Cip1^ positivity: 12.24 ± 9.66% and 13.62 ± 9.74%, respectively, for overall assessed GCNIS), followed by a significant decrease in tumor counterparts. Our finding is in agreement with similar results in pre-malignant conditions and tumor stages of other types of tumors, such as lung adenocarcinoma, classical Hodgkin lymphoma, prostate cancer, and colorectal cancer [12,19,20,21,22]. Also, it complies with the proposed role of senescence as an anti-tumor barrier during the early stages of cancer development that can be elicited by the DNA damage response (DDR) network and factors that regulate the DDR [10,23]. In this frame, the increased immunohistochemical levels in our GCNIS samples of p21^WAF1/Cip1^, a known key downstream effector of p53, is indicative of potential DDR activation [24].

Of note, among all testicular tumor types examined, the highest proportion of senescent cells was identified within the seminomas (Table 2), followed by embryonal carcinomas. Given that the differences between the two histological types were significant, a tempting hypothesis that needs confirmation is that the main anti-tumor response in seminomas is mediated predominantly by senescence activation, while other anti-tumor barriers, like apoptosis, may be more frequently accounted for in the early stages of embryonal carcinoma development [10].

Cellular senescence was also described in the microenvironment of pure seminoma [25]. The authors proposed that the observed senescence may be the cause of the failure of immunotherapy in some patients with seminoma. In our study, testicular cancer was evaluated in samples from patients without any prior treatment. Moreover, while senescent cells were identified in all investigated cancerous histological types, within the seminoma they were more frequent (2.07 ± 1.49% in seminomas versus 0.35 ± 0.18% in embryonal carcinomas, *p* = 0.002) (Table 4). It has been proposed that testicular embryonal carcinomas may represent a successive, more malignant step derived from seminomas [18]. If such a scenario is indeed valid, the decreased frequency of senescent cells may possibly reflect a further breaching of this anti-tumor barrier facilitating disease progression. Nevertheless, such a scenario needs to be mechanistically validated.

Notably, senescence may have a bimodal role in cancer development. On one hand, it acts as a barrier to oncogene-triggered cancer development [10]. On the other hand, any imbalance between induction and elimination of senescent cells may contribute to their abnormal accumulation in various tissues. Such a prolonged presence may have detrimental effects. Particularly in the case of neoplasia, they may foster tumor development either through their persistent SASP, secretory phenotype, or, in a more direct manner, through escape from their growth arrest condition [11]. This cell cycle re-entry has been documented to be associated also with the acquisition of aggressive malignant features [11,26], providing an explanation for the observed tumor recurrences. In our case, it is tempting to speculate that such a mechanism may promote testicular malignancy progression from GCNIS to seminomas and further to embryonic carcinomas. Such scenarios have been documented in Hodgkin lymphomas with the Hodgkin and Reed–Sternberg (HRS) cells, functioning as a pool of senescent cells that drive malignant progression in this disease [12].

To overcome such impediments and increase therapeutic efficacy, the elimination of senescent cells emerges as a potentially promising approach. Given the continuously developing field of senolytics, drugs targeting senescent cells, one may consider more efficient modalities for tumor elimination through the inclusion of such agents in parallel with traditional chemotherapeutic treatments [11].

The introduction of senolytics has been associated with improved outcomes in various cancerous and non-cancerous diseases. The combination of dasanitib and quercetin was applied to patients suffering from diabetic kidney disease in a phase 1 pilot study. The results of skin biopsies, blood examinations, and adipose tissues support the beneficial therapeutic potential of senolytics [27]. Nambiar et al. conducted a phase 1 randomized pilot trial using senolytics in patients with idiopathic pulmonary fibrosis. The authors supported that despite the slightly higher incidence of adverse events, the treatment with senolytics was well tolerated by the patients [28]. A similar study showed that senolytics may lead to ameliorated physical function of the aforementioned patients [29]. There are also promising preclinical studies regarding the utilization of senolytics in cardiovascular and Parkinson’s diseases, and the first clinical trials are ongoing [30,31]. The use of navitoclax was evaluated in terms of the efficacy in eliminating chemotherapy-induced senescence in various different tumors, including melanoma, breast, ovarian, and prostate cancers. The results are very promising, showing the significant perspective of senolytics in the treatment of cancerous diseases [32,33,34,35]. Consequently, a thorough investigation of the role of cellular senescence in testicular cancer may contribute to the presence of senolytics in the therapeutic arsenal.

In most types of testicular cancers, the presence of senescence appears to conform with previously proposed models of cancer development [10,36]. Particularly its presence appears to be high in premalignant lesions, supporting a role as an anti-tumor barrier, while its frequency is low in malignant conditions. Given the potential of senescent cells to contribute to tumor relapses and the advent of senolytic elimination of these cells, classic therapeutic modalities could be revisited to potentially co-include such senescence-targeted treatment for increased efficacy [11] that can be better facilitated at the clinical level by the recent introduction of more effective in vivo senescence-detecting biomarkers [37].

Our study is not without limitations. Firstly, the investigated cohort is relatively small, comprising 30 patients. Despite the low size, it is the first study to our knowledge that investigates cellular senescence status in sequential stages of development of various histological types of testicular cancer. Further investigations with larger sample sizes should be conducted. Moreover, as the outcomes of the immunohistochemical staining were not associated with the pathological stages of cancer and the preoperative tumor markers, further studies should be conducted to obtain deeper insights and clinical outcomes in these neoplastic conditions.

## 5. Conclusions

Cellular senescence is present in testicular tumors, especially in dysplastic (GCNIS) structures. The proportion of senescent cells is predominantly increased in seminomas, followed by embryonal carcinoma, yolk sac tumor, and teratomas, in accordance with the proposed model of testicular cancer development.

## Figures and Tables

**Table 1:** Demographic data, histological proportions, and senescent cells in each histological group. (SD: standard deviation, LVI: lymphovascular invasion/emboli, RTI: rete testis invasion, GCNIS: germ cell neoplasia in situ).

Variable	Outcome
Patients (n)	30
Age (years—mean ± SD)	30.97 ± 8.68
Testis size (cm—mean ± SD)	6 ± 2.80
Tumor size (cm—mean ± SD)	4.16 ± 3.25
Seminoma (%)	13 (43.3%)
LVI (%)	5 (38.5%)
RTI (%)	6 (46.2%)
GCNIS (%)	11 (84.6%)
Embryonal carcinoma (%)	9 (30%)
LVI (%)	6 (66.7%)
RTI (%)	4 (44.4%)
GCNIS (%)	7 (77.7%)
Chondrosarcoma	1 (3.3%)
LVI	No
RTI	No
GCNIS	Yes
Teratoma	4 (13.4%)
LVI (%)	2 (50%)
RTI (%)	1 (25%)
GCNIS (%)	2 (50%)
Yolk sac tumor	3 (10%)
LVI (%)	2 (66.7%)
RTI (%)	1 (33.3%)
GCNIS (%)	2 (66.7%)

**Table 2:** The *p*-values of comparisons between the proportions of p21^WAF1/CIP1^ (+), Ki67+, and GL13(+) cells in different structures. (GCNIS: germ cell neoplasia in situ). The * highlights the statistically significant difference.

Seminoma				
p21^WAF1/Cip1^		Normal	GCNIS	Tumor
	Normal		<0.001 *	0.57
	GCNIS	<0.001 *		<0.001 *
	Tumor	0.57	<0.001 *	
Ki67		Normal	GCNIS	Tumor
	Normal		0.004 *	<0.001 *
	GCNIS	0.004 *		<0.001 *
	Tumor	<0.001 *	<0.001 *	
GL13		Normal	GCNIS	Tumor
	Normal		<0.001 *	0.72
	GCNIS	**<0.001** *		<0.001 *
	Tumor	0.72	<0.001 *	
**Embryonal**				
p21^WAF1/Cip1^		Normal	GCNIS	Tumor
	Normal		0.02 *	0.19
	GCNIS	0.02 *		0.04 *
	Tumor	0.19	0.04 *	
Ki67		Normal	GCNIS	Tumor
	Normal		<0.001 *	<0.001 *
	GCNIS	<0.001 *		<0.001 *
	Tumor	<0.001 *	<0.001 *	
GL13		Normal	GCNIS	Tumor
	Normal		0.03 *	0.06
	GCNIS	0.03 *		0.026 *
	Tumor	0.06	0.026 *	
**Teratoma**				
p21^WAF1/Cip1^		Normal	GCNIS	Tumor
	Normal		0.078	0.45
	GCNIS	0.078		0.011 *
	Tumor	0.45	0.011 *	
Ki67		Normal	GCNIS	Tumor
	Normal		0.059	0.002 *
	GCNIS	0.059		0.007 *
	Tumor	0.002 *	0.007 *	
GL13		Normal	GCNIS	Tumor
	Normal		0.022 *	0.76
	GCNIS	0.022 *		0.08
	Tumor	0.76	0.08	
**Yolk Sac**				
p21^WAF1/Cip1^		Normal	GCNIS	Tumor
	Normal		0.099	0.10
	GCNIS	0.099		0.34
	Tumor	0.10	0.34	
Ki67		Normal	GCNIS	Tumor
	Normal		0.054	0.008 *
	GCNIS	0.054		0.007 *
	Tumor	0.008 *	0.007 *	
GL13		Normal	GCNIS	Tumor
	Normal		0.72	0.62
	GCNIS	0.72		0.19
	Tumor	0.62	0.19	

**Table 3:** Proportions of p21^WAF1/Cip1^ (+), Ki67+, and GL13(+) cells in normal structures, GCNIS, and testicular cancer. Seminoma group. (SD: standard deviation, GCNIS: germ cell neoplasia in situ).

Histological Type	Counterpart	p21^WAF1/CIP1^	Ki67	GL13
Mean ± SD	Normal	2.97 ± 1.06%	2.45 ± 0.89%	1.86 ± 0.84%
	GCNIS	22.68 ± 5.83%	9.56 ± 2.89%	20.81 ± 6.81%
	Tumor	3.32 ± 1.35%	78.08 ± 8.19%	2.07 ± 1.49%

**Table 4:** Proportions of p21^WAF1/Cip1^ (+), Ki67+, and GL13(+) cells in normal structures, GCNIS, and testicular cancer. Embryonal carcinoma group. (SD: standard deviation, GCNIS: germ cell neoplasia in situ).

Histological Type	Counterpart	p21^WAF1/CIP1^	Ki67	GL13
Mean ± SD	Normal	1.76 ± 1.08%	4.23 ± 1.09%	0.47 ± 0.43%
	GCNIS	8.29 ± 6.92%	9.67 ± 2.40%	6.64 ± 5.42%
	Tumor	2.19 ± 1.11%	35 ± 7.77%	0.35 ± 0.18%

**Table 5:** Proportions of p21^WAF1/Cip1^ (+), Ki67+, and GL13(+) cells in normal structures, GCNIS, and testicular cancer. Teratoma group. (SD: standard deviation, GCNIS: germ cell neoplasia in situ).

Histological Type	Counterpart	p21^WAF1/CIP1^	Ki67	GL13
Mean ± SD	Normal	2.13 ± 0.55%	6.53 ± 1.16%	1.15 ± 0.68%
	GCNIS	5.4 ± 2.51%	12.30 ± 3.54%	4.43 ± 1.78%
	Tumor	1.33 ± 0.70%	44.70 ± 7.39%	1.33 ± 0.7%

**Table 6:** Proportions of p21^WAF1/Cip1^ (+), Ki67+, and GL13(+) cells in normal structures, GCNIS, and testicular cancer. Yolk sac group. (SD: standard deviation, GCNIS: germ cell neoplasia in situ).

Histological Type	Counterpart	p21^WAF1/CIP1^	Ki67	GL13
Mean ± SD	Normal	1.27 ± 0.64%	6.33 ± 1.16%	0.77 ± 0.25%
	GCNIS	4.9 ± 1.95%	2.97 ± 1.51%	3.76 ± 1.37%
	Tumor	3.53 ± 0.72%	55 ± 6.56%	1.53 ± 0.55%

**Table 7:** Comparison between seminoma and embryonal carcinoma groups. (SD: standard deviation, LVI: lymphovascular invasion, RTI: rete testis invasion, GCNIS: germ cell neoplasia in situ). The * highlights the statistically significant difference.

Variable	Seminoma	Embryonal Carcinoma	*p* Value
Patients (n)	13	9	
Age (years—mean ± SD)	35 ± 5.97	26.9 ± 10.2	0.053
Testis size (cm—mean ± SD)	4.96 ± 1.21	5.57 ± 2.16	0.46
Tumor size (cm—mean ± SD)	2.9 ± 1.15	3.46 ± 2.67	0.57
LVI (%)	5 (38.5%)	6 (66.7%)	0.19
RTI (%)	6 (46.2%)	4 (44.4%)	0.94
GCNIS (%)	11 (84.6%)	7 (77.7%)	0.17
Normal tissue	1.86 ± 0.84%	0.47 ± 0.43%	<0.001 *
GCNIS	20.82 ± 6.82%	6.64 ± 5.42%	<0.001 *
Tumor	2.07 ± 1.49%	0.35 ± 0.18%	0.002 *

## Data Availability

The data that support the findings of this study are available from the corresponding author upon reasonable request.
